# Prospective Study of Preoperative Negative Affect and Postoperative Pain in Patients Undergoing Thoracic Surgery: The Moderating Role of Sex

**DOI:** 10.3390/jcm13195722

**Published:** 2024-09-25

**Authors:** Angelina R. Franqueiro, Jenna M. Wilson, Jingui He, Desiree R. Azizoddin, Sergey Karamnov, James P. Rathmell, Mieke Soens, Kristin L. Schreiber

**Affiliations:** 1 Department of Anesthesiology, Perioperative and Pain Medicine, Brigham and Women’s Hospital, Boston, MA 02115, USAklschreiber@bwh.harvard.edu (K.L.S.); 2 Department of Family and Preventive Medicine, University of Oklahoma, Oklahoma City, OK 73104, USA; 3 Dana-Farber Cancer Institute, Boston, MA 02115, USA

**Keywords:** negative affect, depression, anxiety, postoperative pain, sex

## Abstract

**Objective:** Preoperative negative affect is a risk factor for worse postoperative pain, but research investigating this association among patients undergoing thoracic surgery is inconsistent. Additionally, female patients often report greater negative affect and postoperative pain than males. This prospective observational study investigated the association between preoperative negative affect and postoperative pain after thoracic surgery and whether this association differed by sex. **Methods:** Patients (n = 105) undergoing thoracic surgery completed preoperative assessments of pain and negative affect (PROMIS anxiety and depression short forms). Patients reported their daily worst pain over the first 7 postoperative days, and an index score of acute postoperative pain was created. Six months after surgery, a subsample of patients (n = 60) reported their worst pain. **Results:** Higher levels of preoperative anxiety (r = 0.25, *p* = 0.011) and depression (r = 0.20, *p* = 0.042) were associated with greater acute postoperative pain, but preoperative negative affect was not related to chronic postsurgical pain (anxiety: r = 0.19, *p* = 0.16; depression: r = −0.01, *p* = 0.94). Moderation analyses revealed that the associations between both preoperative anxiety (b = 0.12, 95% CI [0.04, 0.21], *p* = 0.004) and depression (b = 0.15, 95% CI [0.04, 0.26], *p* = 0.008) with acute postoperative pain were stronger among females than males. Similarly, the association between preoperative anxiety and chronic postsurgical pain was stronger among females (b = 0.11, 95% CI [0.02, 0.20], *p* = 0.022), but the association between preoperative depression and chronic pain did not differ based on sex (b = 0.13, 95% CI [−0.07, 0.34], *p* = 0.201]). **Conclusions:** Our findings suggest that negative affect may be especially important to the experience of pain following thoracic surgery among female patients, whose degree of preoperative anxiety may indicate vulnerability to progress to a chronic pain state. Preoperative interventions aimed at reducing negative affect and pain may be particularly useful among females with high negative affect before thoracic surgery.

## 1. Introduction

Approximately 530,000 thoracic surgeries are performed in the U.S. each year [1], largely to diagnose and/or treat lung cancer, and they include both open thoracotomy and video-assisted thoracoscopic surgery (VATS) [2]. Patients may experience moderate-to-severe acute pain after either surgical technique [3,4], which may predict the development of chronic postsurgical pain [4,5]. Thus, it is important to prospectively investigate factors that may predict worse acute postoperative pain.

The biopsychosocial model of pain outlines a comprehensive set of pain modulators, including psychosocial and biological factors, and it emphasizes how their interactions may contribute to individual differences in the experience of pain [6]. Patients undergoing thoracic surgery commonly report high levels of psychological distress, including depression and anxiety [7,8,9,10]. High levels of preoperative anxiety and depression have both been associated with more severe acute postoperative pain and with the development of chronic postsurgical pain after various procedures [11,12,13,14,15,16,17]. However, research investigating these associations within the context of thoracic surgery is somewhat limited, as it focuses more on chronic postsurgical pain and is inconsistent [4,18]. Some research also suggests that females report greater negative affect than males [14,19], with one study showing that females undergoing thoracic surgery reported higher levels of anxiety than males [7]. Additionally, studies have found that females report more severe pain after surgery, including after thoracic surgery [11,20,21,22,23,24]. Thus, it seems plausible that sex may function as an important moderator in the relationship between preoperative negative affect and postoperative pain severity. However, prior studies have not explored whether sex moderates this relationship in the context of thoracic surgery.

This prospective observational study investigated the association between preoperative negative affect (anxiety, depression) and acute postoperative pain severity among patients undergoing thoracic surgery and evaluated whether this association differed between males and females. We also conducted an exploratory analysis of these associations on chronic pain reported 6 months after surgery among a subgroup of patients who completed assessments at that timepoint. We hypothesized that greater negative affect would be associated with greater postoperative pain and that this association would be stronger among females than males.

## 2. Materials and Methods

### 2.1. Participants and Procedure

**Figure 1:**
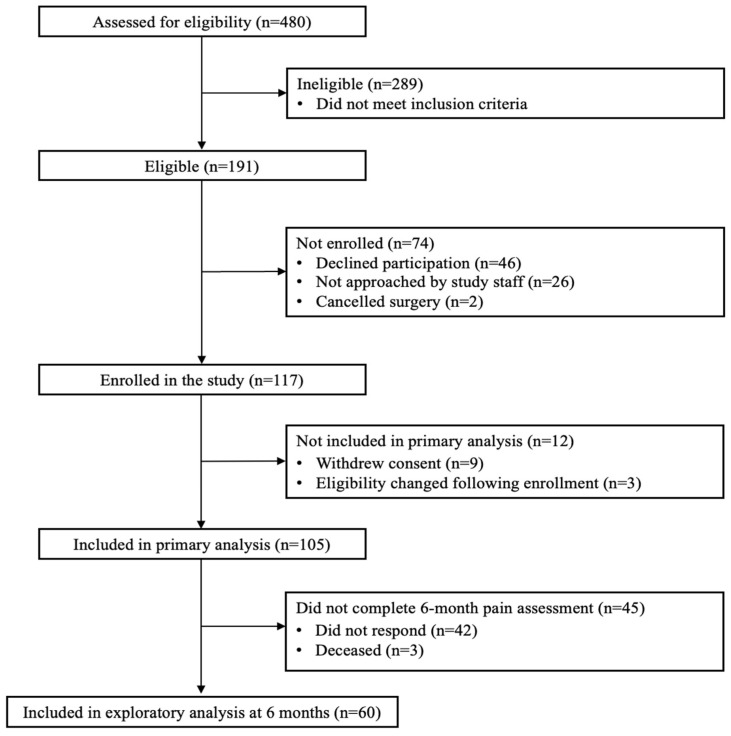
Study flow diagram.

This prospective observational study recruited patients 18–85 years of age scheduled for thoracic surgery at Brigham and Women’s Hospital, a large tertiary referral hospital in Boston, MA. Data were collected from January 2018–June 2022. Eligible patients were recruited via referral from the operating surgeon, informational flyers, and phone calls from the study staff. In order to reduce confounding effects due to pre-existing pain and pain-related factors, patients were excluded if they met any of the following criteria: (1) pre-existing chronic pain condition or opioid use; (2) current treatment with corticosteroids; (3) evidence of an active infection; (4) chronic liver disease, end-stage renal disease, or chronic inflammatory disorders; (5) recent major surgery or illness within 30 days; (6) use of immunosuppressive medication; and/or (7) history of organ transplantation (Figure 1). After providing informed consent, patients completed preoperative questionnaires assessing demographic, psychological, and pain characteristics. After surgery, patients completed daily pain assessments during the first postoperative week (postoperative days 1–7). Patients reported daily pain scores to research staff once per day prior to discharge. After discharge, patients reported daily pain scores via online surveys sent as SMS text messages through REDCap [25,26]. The Partners Human Research Committee/Institutional Review Board approved all study procedures.

### 2.2. Measures

#### 2.2.1. Preoperative Assessments

Prior to surgery, patients self-reported their maximum (worst) pain experienced over the past 24 h on a scale of 0 (no pain) to 10 (worst pain imaginable). The Patient-Reported Outcomes Measurement Information System (PROMIS) [27] short forms have been validated in adult patients across a variety of samples and were used to measure patients’ anxiety and depression over the past week. Seven items assessed anxiety (e.g., “I felt uneasy”) (α = 0.94) and eight items assessed depression (e.g., “I felt worthless”) (α = 0.91) on a scale of 1 (never) to 5 (always). Items were summed, and higher scores indicated greater anxiety (range: 7–35) and depression (range: 8–40), respectively. Using the PROMIS score conversion table, raw scores were converted to standardized T-scores with a mean of 50 and a standard deviation (SD) of 10 [27]. Additionally, patients reported demographic information, including age, sex, race/ethnicity, and weight and height to calculate body mass index (BMI). Patients’ medical history (e.g., current smoking and/or use of tobacco, prior chest surgery, preoperative cancer diagnosis) was extracted from the electronic medical record.

#### 2.2.2. Acute Postoperative Assessments

Across postoperative days (PODs) 1–7, patients reported their daily maximum pain severity experienced over the past 24 h (0 = no pain, 10 = worst pain imaginable), either verbally to study staff (while inpatient) or via secure text message (after discharge). All 7 maximum pain ratings were averaged to create an index score (α = 0.92) of acute postoperative pain severity. Clinical information (e.g., postoperative complications, subsequent diagnosis of cancer) was extracted from the electronic medical record.

#### 2.2.3. Chronic Postsurgical Pain

Patients completed the Brief Pain Inventory [28], a validated assessment of chronic pain, at 6 months after surgery, which included a 1-item assessment of worst pain severity experienced over the past week (0 = no pain, 10 = worst pain imaginable).

### 2.3. Data Analysis

Demographic, clinical, psychosocial, and pain characteristics were reported using basic descriptive statistics, with continuous data presented as means and standard deviations (SDs) and categorical data presented as percentages. Bivariate correlations were used to examine the associations between preoperative anxiety and depression with acute and chronic postoperative pain. The Mann–Whitney U and chi-square tests were used to investigate whether demographic and clinical characteristics, negative affect, or pain assessments differed between males and females. Four moderation analyses were conducted to examine whether the associations between preoperative anxiety and depression with postoperative pain severity differed between males and females using the PROCESS macro for IBM SPSS Statistics for Macintosh, version 29.0 (IBM Corp., Armonk, NY, USA) [29]. In each model, preoperative negative affect (anxiety, depression) was entered as the independent variable (x), postoperative pain as the dependent variable (y), and sex as a moderator (m). Bias-corrected 95% confidence intervals (CI) were created based on 5000 bootstrapped resamples. A significant interaction was followed up with post hoc probing. A *p*-value ≤ 0.05 was the threshold for significance for all calculations. Additionally, we conducted a post hoc power analysis, which showed that our sample of 105 participants could detect a small- to medium-sized effect (f^2^ = 0.09) of an interaction (moderation), assuming a power of 0.80 and α = 0.05.

## 3. Results

### 3.1. Preoperative Patient Characteristics

Patients (N = 105) had an average age of 64.9 years (SD = 10.8; range: 35–83), an average BMI of 27.9 (SD = 5.2; range: 17.4–41.6), and 57% were female. The majority of patients identified as White (95%), with others identifying as Hispanic/Latino (2%), Asian (1%), Hawaiian or Pacific Islander (1%), or Mixed Race (1%). Of all patients, 6% reported currently smoking or using tobacco. Prior to surgery, 25.7% of patients had been diagnosed with cancer, 3.8% had a non-cancerous indication for surgery (e.g., emphysema), and for the majority of patients (70.5%), the presence of cancer was unknown at the time of surgery. There were no significant sex differences in any preoperative patient characteristics (Table 1).

**Figure 2:**
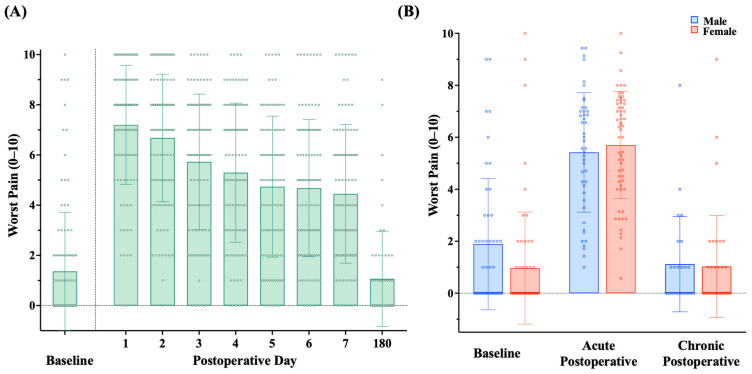
Self-reported maximum pain severity scores at baseline (before surgery), across postoperative days 1–7, and on postoperative day 180 (6 months postoperative) for the entire sample are shown in (**A**). Self-reported maximum pain severity at baseline, across the first postoperative week (acute), and at 6 months postoperative (chronic) for male (blue) and female (red) patients is shown in (**B**). The acute postoperative pain scores in (**B**) are the mean of patients’ daily maximum pain scores across postoperative days 1–7. Bars represent the mean maximum pain scores, and error bars represent the standard deviation from the mean. Circles denote individual patient pain scores at each timepoint.

Patients reported relatively little preoperative pain, with a maximum pain score of 1.4 (SD = 2.4; range: 0–10) prior to surgery (Figure 2A). A significantly higher maximum pain score was seen among males (M = 1.9, SD = 2.5) compared to females (M = 1.0, SD = 2.2) (*p* = 0.011) at baseline (Figure 2B). Patients reported a range of anxiety and depression prior to surgery, with average T-scores of 52.8 (SD = 9.9, range: 36.3–82.7) for anxiety and 45.9 (SD = 8.3, range: 37.1–67.4) for depression. There were no significant sex differences in preoperative anxiety (males: M = 51.6, SD = 8.8 vs. females: M = 53.7, SD = 10.6) or depression (males: M = 46.0, SD = 8.4 vs. females: M = 45.8, SD = 8.3) in our patient sample (Supplemental Figure S1).

### 3.2. Surgical Characteristics

The majority (86%) of patients underwent video-assisted thoracoscopic surgery (VATS), with 63% undergoing a smaller surgical procedure (e.g., VAT segmentectomy or wedge resection) and 23% undergoing a larger procedure (e.g., VAT lobectomy or pleurectomy), while 14% underwent open thoracotomy (10 standard, 4 mini-thoracotomy). Previous chest surgery was reported in 43% of patients, with 20% having had surgery on the same surgical side. On average, patients stayed in the hospital for 3.6 days (SD = 2.9; range: 1–15), and 29% experienced some sort of postsurgical complication (predominantly a need for a prolonged chest tube due to an air leak and, more rarely, treatment for suspected pneumonia [n = 2], atrial fibrillation with rapid ventricular response [n = 3], and hemothorax or pleural effusion requiring a return to the operating room [n = 2]). Patients had either one (86%) or two (14%) chest tubes placed. Of the patients who had a chest tube placed, the majority had a 24 French (49%), 32% had a 28 French, 18% had a 19 French, and 1% had a 14 French. Chest tubes remained in place for an average of 3.3 days (SD = 3.6; range: 1–21). More than half (57%) of the patients were diagnosed with cancer as a result of tissue collected at the time of surgery. No surgical characteristics were significantly associated with the severity of acute postoperative pain (Table 2). Similarly, no sex differences were seen in the surgical characteristics (Table 1).

### 3.3. Association of Preoperative Negative Affect with Acute Postoperative Pain: The Role of Sex

After surgery, the patients reported an average maximum acute postoperative pain severity score of 5.6 (SD = 2.2) across PODs 1–7, with considerable interindividual variability (Figure 2A). While we did not observe a significant difference in acute postoperative pain between males (M = 5.4, SD = 2.3) and females (M = 5.7, SD = 2.1) (Figure 2B), 63% of females vs. 37% of males experienced severe pain (mean maximum pain score ≥ 7) in the first week after surgery. Across the entire group, there were modest associations of preoperative anxiety (r = 0.25, *p* = 0.011) and depression (r = 0.20, *p* = 0.042) with acute postoperative pain, such that higher preoperative levels of both anxiety and depression were associated with greater maximal acute postoperative pain severity.

**Figure 3:**
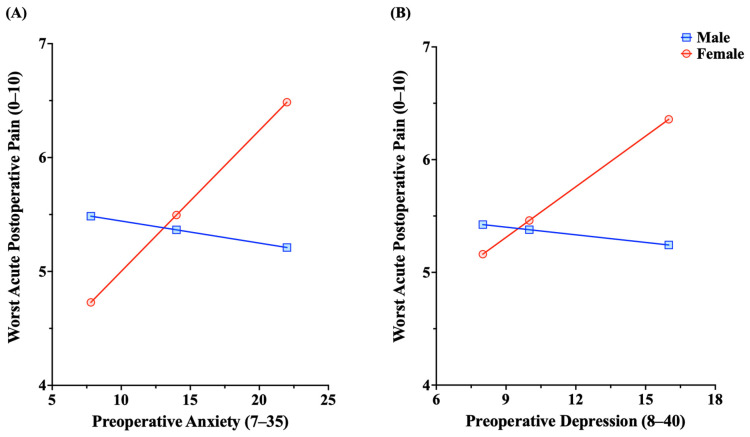
The moderating role of sex in the association between acute postoperative pain severity and (**A**) preoperative anxiety and (**B**) preoperative depression. For females (red line), a stronger association was observed than for males (blue line).

To assess whether the associations of preoperative depression and anxiety with acute postoperative pain differed based on sex, we performed moderation analyses. The model with preoperative anxiety and sex predicting acute postoperative while controlling for baseline pain severity accounted for 7.4% of the variance in postoperative pain. We then observed a significant interaction between preoperative anxiety and sex (b = 0.14, 95% CI [0.004, 0.28], *p* = 0.044) (Figure 3A). We found that, among females, higher preoperative levels of anxiety were significantly associated with greater acute postoperative maximum pain severity (b = 0.12, 95% CI [0.04, 0.21], *p* = 0.004), while this was not the case among males (b = −0.02, 95% CI [−0.13, 0.10], *p* = 0.739). Adding this interaction to the model accounted for an additional 3.7% of variance in acute postoperative pain. We then observed a significant interaction between preoperative depression and sex (b = 0.17, 95% CI [0.01, 0.34], *p* = 0.043) while controlling for baseline pain severity (Figure 3B). Again, among females, higher preoperative levels of depression were significantly associated with greater acute postoperative maximum pain severity (b = 0.15, 95% CI [0.04, 0.26], *p* = 0.008), while this was not the case among males (b = −0.02, 95% CI [−0.15, 0.11], *p* = 0.726). Adding this interaction to the model accounted for 2.9% of the variance in acute postoperative pain.

### 3.4. Exploratory Analysis: Preoperative Negative Affect, Chronic Postsurgical Pain, and Sex

Six months after surgery, patients completed a follow-up assessment of their worst pain severity experienced over the past week as a measure of chronic postsurgical pain. Of the entire sample (n = 105), 60 patients (57% of the sample; 25 males, 35 females) completed this follow-up assessment. Patients who completed the 6-month follow-up pain assessment (n = 60) did not significantly differ from patients who did not complete the follow-up assessment (n = 45) based on demographic (age, sex, race/ethnicity), clinical (cancer diagnosis), or surgical (type of surgery, length of stay, postoperative complications) characteristics (*p* > 0.05).

Of this subsample, only five patients (8%) reported a chronic pain score ≥ 4/10, with many patients endorsing little-to-no pain (M = 1.1, SD = 1.9, range: 0–9) (Figure 2A). We did not observe a significant difference in chronic postsurgical pain between males (M = 1.1, SD = 1.8) and females (M = 1.0, SD = 2.0) (Figure 2B). Among this subsample, there were no significant associations between preoperative anxiety (r = 0.19, *p* = 0.16) or depression (r = −0.01, *p* = 0.94) with chronic pain severity.

To parallel the acute postoperative pain analysis, we explored whether the associations of preoperative anxiety and depression with chronic postsurgical pain differed between males and females. A moderation analysis showed a marginally significant interaction between preoperative anxiety and sex on chronic postsurgical pain (b = 0.15, 95% CI [−0.003, 0.30], *p* = 0.055). Similar to the pattern observed with acute postoperative pain, we found that, among females, higher preoperative levels of anxiety were significantly associated with greater chronic postsurgical pain severity (b = 0.11, 95% CI [0.02, 0.20], *p* = 0.022), while this was not the case among males (b = −0.04, 95% CI [−0.16, 0.08], *p* = 0.504). However, there was not a significant interaction between preoperative depression and sex (b = 0.13, 95% CI [−0.07, 0.34], *p* = 0.201). Thus, the association between preoperative anxiety and chronic postsurgical pain severity was stronger among females than males, whereas the association between preoperative depression and chronic postsurgical pain did not differ based upon sex.

## 4. Discussion

The present study investigated the relationship between preoperative negative affect and postoperative pain severity and whether this relationship differed based on sex in patients undergoing thoracic surgery. Higher preoperative levels of both anxiety and depression were associated with greater acute postoperative pain severity during the first week following thoracic surgery. While preoperative negative affect and acute postoperative pain did not differ by sex, moderation analyses revealed that the associations between preoperative negative affect and acute postoperative pain were stronger among females than males. Specifically, higher preoperative levels of anxiety and depression were each associated with worse acute postoperative pain severity among females but not males. These findings highlight the importance of considering sex in relation to the influence of preoperative negative affect on postoperative pain.

Preoperative depression and anxiety have shown to be associated with worse acute and chronic postoperative pain in other surgical cohorts, including mastectomy (female only), total knee arthroplasty (male and female), and cesarean delivery (female only) [13,14,30]. In thoracic surgical cohorts, work focusing on predictors of acute postoperative pain is somewhat limited, and the association between negative affect and pain is less consistent. One previous study focusing on acute postsurgical pain found that preoperative levels of anxiety and depression did not differ between patients with moderate–severe acute postoperative pain (pain score ≥ 4/10) and those with mild acute pain (pain score < 4/10) [18]. This contrasts our finding that higher preoperative levels of anxiety and depression were both associated with worse acute pain during the first postoperative week. For chronic pain after thoracic surgery, several studies have not found evidence of a significant association between preoperative anxiety or depression with postsurgical pain [31,32,33,34,35], whereas other studies have shown a significant positive association [15,36]. In the present study, we did not observe a significant association between preoperative anxiety or depression with chronic postsurgical pain. The definition of chronic pain, the inclusion of patients with existing chronic pain, and the sample size may all potentially contribute to whether significant associations are observed, thus contributing to variability among study findings.

In the present study, which excluded patients with a chronic pain diagnosis, patients reported relatively low levels of both acute and chronic postoperative pain, and we did not observe a significant difference in acute or chronic postsurgical pain severity between males and females. Our findings are aligned with some studies that have reported similar levels of acute pain among males and females after thoracic surgery [3,37], although these findings contrast studies that suggest that females are at greater risk for worse acute postoperative pain compared to males [21,38]. Our findings also contrast a recent meta-analysis that showed that females tend to have a higher risk of developing chronic postsurgical pain after thoracic surgery [39]. While we found that a higher percentage of females (63%) reported severe postsurgical pain (≥7/10) than males (37%), this was not statistically significant, and it is possible that the relatively low levels of pain reported by patients in the present study prohibited our ability to detect sex differences.

Previous work, the majority of which has been conducted in other surgical cohorts, has suggested that females report higher levels of negative affect, a psychological risk factor for greater postoperative pain, than males. In the present study, however, we did not observe a significant difference in preoperative anxiety or depression between females and males, which contrasts one prior study that found that females undergoing thoracic surgery reported higher preoperative levels of anxiety than males [7]. Several studies have demonstrated that individuals older in age report lower levels of negative affect, including both anxiety and depression, and pain severity than those younger in age [40,41,42]. As our sample was relatively older in age compared to prior studies, it is plausible that this may have contributed to the observed lower levels of and lack of observable sex differences in preoperative negative affect and postoperative pain.

Notably, we found that sex significantly moderated the association between preoperative negative affect and postoperative pain severity, such that the associations between preoperative levels of anxiety and depression with postoperative pain severity were stronger among females compared to males. The mechanisms underlying this interaction are not elucidated by our design but might speculatively include a relatively greater activation of pain by pre-existing anxiety and depression among females due to a greater cultural acceptability of expression of negative affect in women than men. It is possible that behavioral interventions, such as cognitive behavioral therapy (CBT) and Mindfulness-Based Stress Reduction (MBSR), which have been shown to effectively reduce both negative affect and pain severity among patients undergoing surgery [43,44], may be particularly beneficial for females. A recent systematic review of randomized controlled trials (RCTs) implementing cognitive behavioral strategies (e.g., cognitive restructuring, behavioral self-management) perioperatively showed evidence that these strategies effectively reduce postoperative pain [45]. Additionally, in at least one study in patients with chronic pain, females reported greater improvement in pain severity compared to males following a behavioral intervention [46], although sex differences in treatment response are variable across studies [47].

Sex differences in other postsurgical outcomes have been observed within the literature, including surgical site infection [48,49], need for hospital readmission [50], reoperation [51], and overall mortality [52], favoring males. One speculative explanation for these sex-based disparities is a perioperative change in hormonal levels that females undergoing surgery may experience. While estrogen has been reported to exhibit cardioprotective effects by mitigating endothelial dysfunction [53,54] and promoting adaptive immunity by impacting levels of circulating antibodies [55] under normal conditions, the experience of surgical stress and general anesthesia have been associated with decreased estrogen levels, thereby disrupting the baseline state of regulation in females [56]. Within the context of pain, sudden fluctuations in estrogen levels have been associated with greater pain sensitivity [57]. Fluctuations in estrogen levels have also been associated with greater anxiety and depressive symptoms [58], which may influence pain in the perioperative period. Fluctuations in pain, in the perioperative context, may in turn trigger further increases in anxiety, potentially activating a negative spiral in patients who are predisposed to higher preoperative negative affect. Such interactions are speculative, and future prospective and well-controlled studies are needed to examine the relevance of fluctuations in estrogen plasma levels to anxiety, postsurgical pain, and their interaction.

There are several limitations that must be considered when interpreting the present findings. First, the preoperative and postoperative pain severity scores were fairly low in our sample and may not reflect general samples, which typically include patients with chronic pain conditions and may have a greater propensity to suffer worse postsurgical pain. Second, the racial/ethnic diversity of our sample was quite limited. As the majority of patients in the present study identified as White, our findings may not generalize to a more diverse racial/ethnic sample, in which the experience of postoperative pain may differ. Several studies in surgical cohorts have shown that Black and Indigenous People of Color (BIPOC) patients report greater postoperative pain severity than White patients [59,60,61,62]. Future work should investigate whether racial and ethnic disparities in postoperative pain are also observed in the context of thoracic surgery. Potentially, associations between preoperative negative affect and postoperative pain may differ in a more diverse sample. Third, we assessed patient sex, not gender, as a moderator of the association between preoperative negative affect and postoperative pain. Future studies should measure both sex and gender, such that both can be examined as potential moderators. This could further identify targets to tailor prospective interventions for postoperative pain. Finally, the present study was conducted at a large academic medical institution, and our results may not generalize to institutions in different regions.

## 5. Conclusions

The present study examined the association between preoperative negative affect and postoperative pain among patients undergoing thoracic surgery and whether sex moderated this association. We found significant associations between both preoperative anxiety and depression with greater maximal acute pain severity during the first week after surgery, and significant moderating effects suggested that these associations were stronger among females than males. However, we did not find a significant association between preoperative anxiety or depression with chronic postsurgical pain 6 months after surgery. Further, the association of preoperative anxiety with greater chronic pain severity was stronger among females than males, whereas the association of preoperative depression with chronic pain severity did not differ between males and females.

## 6. Clinical Implications

Our findings suggest that negative affect, particularly anxiety, is important to the experience of postsurgical pain following thoracic surgery among females. This may have several important implications for personalizing perioperative care. Potentially, preoperative interventions intended to reduce both negative affect and pain, such as CBT or MBSR, could be especially useful among females with high levels of negative affect before thoracic surgery. Future RCTs targeting negative affect should assess potential moderators of their efficacy, taking both sex and baseline negative affect into account as potential effect modifiers.

## Figures and Tables

**Table 1:** Sex differences in patient and surgical characteristics.

	Males (n = 45)	Females (n = 60)	
	Mean ± SD or n (%)	Mean ± SD or n (%)	*p*-Value
Age (n = 105)	67.0 ± 9.2	63.3 ± 11.7	0.15
BMI (n = 105)	28.3 ± 4.9	27.6 ± 5.5	0.37
White race (n = 105)	43 (96%)	57 (95%)	0.90
Currently smoking/tobacco use (n = 105)	2 (4%)	4 (7%)	0.63
Preoperative cancer diagnosis (n = 105)			0.93
No	2 (4.4%)	2 (3%)	
Yes	11 (24.4%)	16 (27%)	
Unknown	32 (71.1%)	42 (70%)	
Had prior chest surgery on same side (n = 99)	6 (14%)	14 (25%)	0.18
Type of surgery (n = 105)			0.34
Minor VATS surgery (e.g., biopsy, wedge resection)	26 (58%)	40 (67%)	
Major VATS surgery (e.g., VATS segmentectomy)	10 (22%)	14 (23%)	
Thoracotomy	9 (20%)	6 (10%)	
More than 1 chest tube (n = 104)	9 (20%)	6 (10%)	0.16
Size of chest tube (n = 97)			0.57
14		1 (2%)	
19	6 (14%)	11 (20%)	
24	21 (49%)	27 (50%)	
28	16 (37%)	15 (28%)	
Had postoperative complications (n = 105)	16 (36%)	14 (23%)	0.17
Length of stay (n = 105)	3.9 ± 3.1	3.3 ± 2.8	0.21

Note. Sample sizes reflect the total number of patients with complete data for each variable.

**Table 2:** Associations of patient and surgical characteristics with acute postoperative pain.

	Acute Pain Severity	*p*-Value
Age (n = 105)	−0.05	0.63
BMI (n = 105)	−0.09	0.39
Race (n = 105)		0.54
White	5.61 ± 2.18	
Non-White	4.10 ± 1.73	
Current smoking/tobacco use (n = 105)		0.12
No	5.50 ± 2.17	
Yes	6.93 ± 1.59	
Preoperative cancer diagnosis (n = 105)		0.17
No	6.86 ± 1.91	
Yes	6.05 ± 1.79	
Unknown	5.34 ± 2.27	
Prior chest surgery, same side (n = 99)		0.93
No	5.62 ± 2.16	
Yes	5.67 ± 2.20	
Type of surgery (n = 105)		0.35
Minor VATS surgery (e.g., biopsy, wedge resection)	5.67 ± 2.23	
Major VATS surgery (e.g., VATS segmentectomy)	5.06 ± 2.13	
Thoracotomy	6.03 ± 1.88	
Number of chest tubes (n = 104)		0.39
1	5.50 ± 2.24	
2	6.02 ± 1.66	
Size of chest tube (n = 97)		0.16
14	4.2 ± 3.3	
19	5.8 ± 2.0	
24	5.2 ± 2.3	
28	5.4 ± 2.3	
Postoperative complications (n = 105)		0.41
No	5.58 ± 2.13	
Yes	6.39 ± 2.49	
Length of stay (n = 105)	0.14	0.17

Note. Bivariate correlations for continuous variables. Chi-square tests for categorical variables. Postoperative complications within the first week predominantly included need for prolonged chest tube due to air leak and, more rarely, treatment for suspected pneumonia (n = 2), atrial fibrillation with rapid ventricular response (n = 3), and hemothorax or pleural effusion requiring return to the operating room (n = 2).

## Data Availability

Data are available from the authors upon request.

## Supplementary Materials

The following supporting information can be downloaded at: https://www.mdpi.com/article/10.3390/jcm13195722/s1, Supplemental Figure S1: Preoperative Anxiety and Depression.
