# Supplementary figures and images for: Prospective Study of Preoperative Negative Affect and Postoperative Pain in Patients Undergoing Thoracic Surgery: The Moderating Role of Sex
Source: J Clin Med. 2024 Sep 25;13(19):5722. doi: 10.3390/jcm13195722 (PMC11476742; doi:10.3390/jcm13195722)

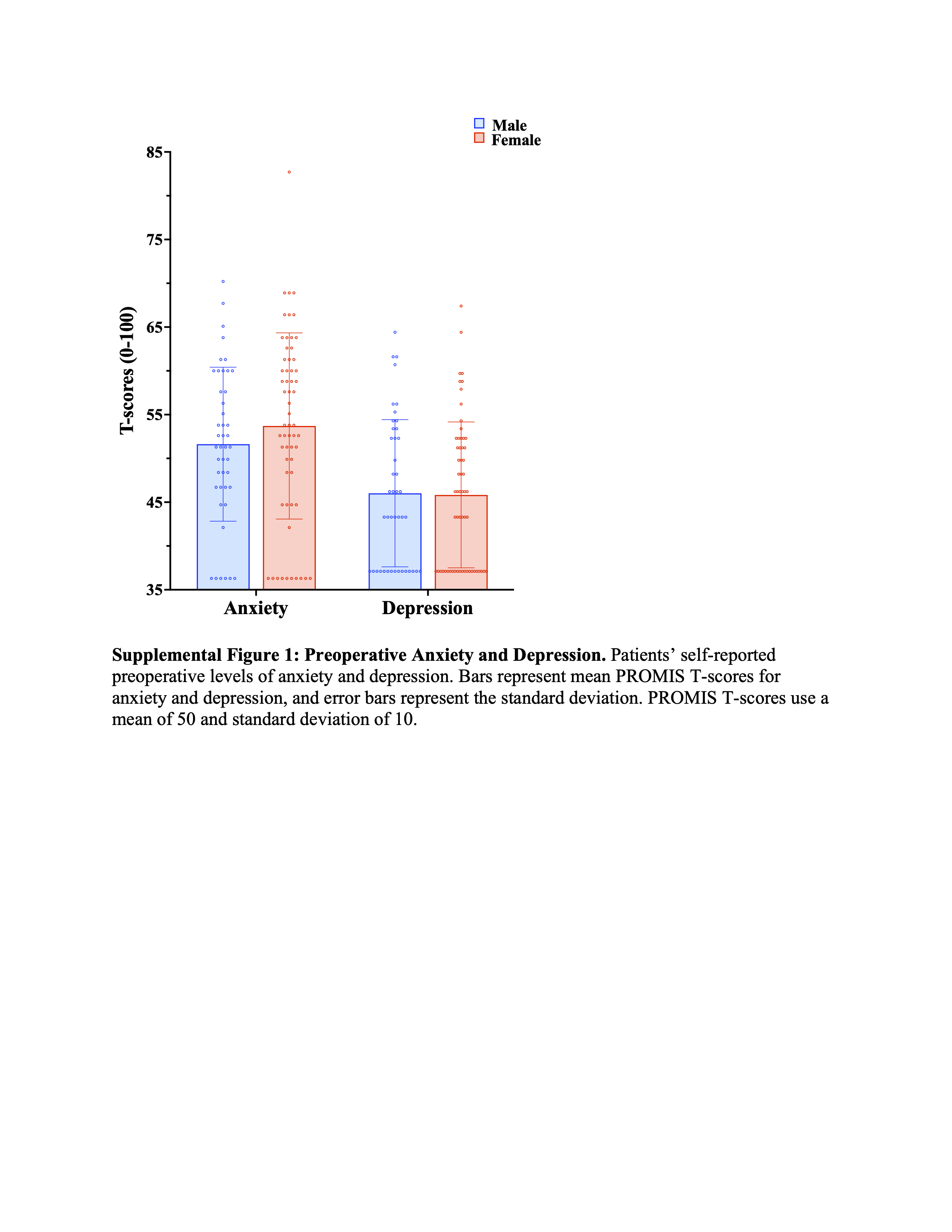

Supplement: Supplementary file 1 [file jcm-13-05722-s001.zip › jcm-3184822-supplementary.png]
